# Near-Total Pancreatectomy for Congenital Hyperinsulinemic Hypoglycemia: A Single-Center Experience From a Low-Resource Setting in Sudan

**DOI:** 10.7759/cureus.111410

**Published:** 2026-06-24

**Authors:** Isam Ahmed Abdeljaleel Taha, Mohamed A Helali, Sami Mohamed Elamin Taha, Musab Mohammed, Mohamed Y Ibrahim, Elsiddig Yagoub, Ishag Nadi Joseph, Nuseiba Abdo, Hayat Abuobaida Banagh, Basma Suliman, Mohamed A Ali, Ahmed M Osman

**Affiliations:** 1 Pediatric Surgery, Pediatric Surgery Center/National Ribat University Hospital, Khartoum, SDN; 2 Pediatric Surgery, National Ribat University, Khartoum, SDN; 3 Pediatric Surgery, Dr. Mohamed Yahia Clinic, Khartoum, SDN; 4 Pediatric Surgery, National Ribat University Hospital, Khartoum, SDN

**Keywords:** congenital hyperinsulinism, diazoxide‑unresponsive hyperinsulinism, near-total pancreatectomy, neonatal hypoglycemia, pancreatic surgery in infants, persistent hypoglycemia

## Abstract

Background and objective

Congenital hyperinsulinemic hypoglycemia (CHH) is the leading cause of persistent hypoglycemia in neonates and infants. In high-resource settings, diagnosis and management depend on rapid genetic testing and advanced imaging to differentiate focal from diffuse disease. However, in low-resource settings, limited diagnostic capacity and lack of access to medications often necessitate early surgical intervention. This study aimed to evaluate the clinical features, surgical intervention, and outcomes in infants undergoing pancreatectomy for congenital hyperinsulinism at Al Ribat University Hospital, Sudan.

Methods

A retrospective descriptive study was conducted, involving all pediatric patients who underwent pancreatectomy for CHH from January 2019 to December 2024. Data were extracted from operative logs and medical records, including demographics, diagnostic investigations, medical therapy, surgical procedures, postoperative complications, histopathology, and one-year outcomes. Descriptive statistics and appropriate inferential statistical tests were used for the analysis.

Results

Thirty-two patients were included, of whom 19 (59.4%) were female. The majority were diagnosed during the first week of life (n = 19, 59.4%). Diazoxide was administered to 30 (93.75%) patients, with only eight (26.7%) showing a therapeutic response. Restricted access to genetic testing (n = 8, 25%) and imaging (n = 6, 18.8%) resulted in the predominance of near-total pancreatectomy (n = 31, 96.9%). Intraoperative complications were minimal, with bleeding occurring in two cases (6.25%) and vascular injury in one case (3.1%). Early postoperative hypoglycemia was noted in 13 patients (40.6%), whereas hyperglycemia was noted in one patient (3.1%). Surgical complications included wound infection in seven patients (21.9%) and intestinal obstruction in one patient (3.1%). Histopathology confirmed CHH in all cases, with diffuse disease in 27 patients (84.4%). During a one-year follow-up period, three patients (9.4%) died, including two (6.3%) due to recurrent hypoglycemia.

Conclusions

Near-total pancreatectomy remains a practical and life-saving option for infants with medically unresponsive CHH in low-resource settings where diagnostic and pharmacologic limitations restrict individualized care. Despite limited access to preoperative localization and the predominance of diffuse disease, surgical outcomes were acceptable, with low intraoperative morbidity and manageable rates of postoperative complications. Persistent postoperative hypoglycemia and late mortality highlight the need for structured endocrine follow-up and improved access to medical therapy. Strengthening diagnostic capacity, ensuring medication availability, and establishing coordinated multidisciplinary care pathways are essential to improving long-term outcomes for children with CHH in resource-limited environments.

## Introduction

Congenital hyperinsulinemic hypoglycemia (CHH) is the most common cause of persistent hypoglycemia in neonates and infants, resulting from dysregulated insulin secretion despite low blood glucose levels [1]. Prompt and effective management is crucial, as recurrent or prolonged episodes of hypoglycemia can lead to severe neurological injury, long-term cognitive impairment, and developmental delay [2]. CHH can occur in both focal and diffuse forms, which reflects underlying genetic and histopathological differences that influence management decisions [3].

The diagnosis of CHH requires a structured and prompt approach to confirm inappropriate insulin secretion during hypoglycemia and to distinguish between focal and diffuse disease [3]. Recent international guidelines recommend collecting a blood sample during spontaneous or induced hypoglycemia, demonstrating inappropriately elevated insulin, low free fatty acids, suppressed ketone bodies, and a positive glycemic response to glucagon [2]. Rapid genetic testing is highly recommended as a first-line diagnostic tool, as mutations in ABCC8 and KCNJ11 genes not only confirm the diagnosis but also help predict diazoxide responsiveness and guide surgical management [1,4]. When available, 18F-DOPA PET/CT is considered the gold standard imaging modality for differentiating focal from diffuse CHH, enabling curative focal lesionectomy and avoiding unnecessary near-total pancreatectomy. These standardized diagnostic pathways significantly improve diagnostic accuracy and clinical outcomes [5].

Medical management is considered the first-line approach, including concentrated glucose infusion and pharmacologic agents such as diazoxide and octreotide. Many infants with the diffuse form do not respond to maximal medical therapy, requiring progression to surgical intervention; nevertheless, diazoxide remains the first-line pharmacologic treatment [6]. Current consensus recommendations emphasize the need for standardized, multidisciplinary pathways in the diagnosis and management of CHH. The 2023 UK national collaborative consensus highlighted the importance of structured biochemical evaluation, rapid genetic testing, and the integration of advanced imaging modalities to accurately differentiate focal from diffuse disease and guide surgical planning.

These latest guidelines highlight how coordinated, protocol-driven care can markedly enhance clinical outcomes; however, they also underscore the challenges faced in regions where such diagnostic resources remain limited [5]. In resource-limited settings with limited access to advanced imaging techniques and comprehensive genetic testing, pancreatectomy, particularly near-total resection, is often required for medically unresponsive cases. Nevertheless, surgery is associated with significant risks, including recurrent hypoglycemia, pancreatic insufficiency, persistent hypoglycemia, and the long-term development of diabetes mellitus [7,8]. Outcomes vary markedly between centers and are influenced by diagnostic capacity, availability of genetic testing, expertise in pediatric pancreatic surgery, and overall healthcare system infrastructure [9]. In low-resource settings, additional challenges such as delayed referral, delayed diagnosis, and medication shortages may further affect management decisions and patient outcomes [10].

To the best of our knowledge, this study represents the first documented Sudanese series to evaluate surgical management, specifically near-total pancreatectomy, for CHH. No prior Sudan-based research addressing CHH diagnosis, limitations in medical therapy, or surgical outcomes has been published. A focused search of the African literature revealed only isolated case reports, such as a single Nigerian infant with CHH reported in 2023, but no multicenter or country-level African studies describing surgical cohorts, pancreatectomy outcomes, or CHH management pathways. While a significant evidence gap exists across the continent, CHH remains underreported despite substantial diagnostic and therapeutic challenges. Therefore, our study provides the first regional dataset from Sudan and one of the very few contributions from Africa, offering valuable insights into CHH care in a low-resource setting [11].

This study aims to describe the clinical characteristics and surgical outcomes in infants with CHH undergoing near-total pancreatectomy in a low-resource setting. Secondary observations focus on challenges related to diagnosis and perioperative management in the absence of advanced imaging modalities.

## Materials and methods

Study design and setting

This was a retrospective descriptive study conducted at the Department of Pediatric Surgery, Al Ribat University Hospital, Sudan. This study aimed to evaluate the clinical features, surgical treatment options, and outcomes of pancreatectomy conducted for CHH over five years from January 2019 to December 2024.

Study population and sample size

The study included all pediatric patients diagnosed with CHH who underwent pancreatectomy at Al Ribat University Hospital during the study period. A total of 32 patients met the inclusion criteria and were included in the analysis. The study population consisted mainly of neonates and infants who underwent pancreatic surgery for CHH. Patients with incomplete medical records or those who underwent pancreatic surgery for indications other than CHH were excluded from the study.

Data collection

Data were collected retrospectively from hospital medical records, operative theater logbooks, inpatient files, and available electronic databases. Extracted variables included patient demographics (age and gender), age at diagnosis of hypoglycemia, family history of CHH, referral source, preoperative medical management (including use of dextrose infusion and diazoxide and response to therapy), diagnostic investigations performed, and indication for surgery. Operative data included age at surgery, type of pancreatectomy performed, and intraoperative complications such as bleeding, injury to the adjacent structures, and intraoperative mortality. Postoperative data included early postoperative glucose control, surgical complications, timing of initiation of oral feeding, histopathological findings, and mortality during one-year follow-up.

Clinical definitions and operational criteria

Urgent surgery was defined as an operative intervention performed within 24 hours of admission in cases where hypoglycemia remained refractory to maximal medical therapy, including escalation of glucose infusion rate (GIR), high‑concentration dextrose administration, and optimized supportive measures. Urgency was determined when persistent hypoglycemia posed an immediate risk of neurological injury. Diazoxide responsiveness was defined as the achievement of stable euglycemia (blood glucose ≥70 mg/dL) without the need for intravenous glucose infusion after titration of diazoxide up to 15 mg/kg/day for a minimum of five days. Patients who continued to require intravenous glucose or failed to maintain euglycemia were classified as non‑responsive. Postoperative hypoglycemia was defined as any documented blood glucose value <70 mg/dL within the first 48 hours following surgery, based on serial point‑of‑care monitoring.

Data analysis

Descriptive statistical analysis was performed using Microsoft Excel. Categorical variables were summarized as frequencies and percentages, while continuous variables were described using means or medians as appropriate. Results were organized into tables for clarity and comparison.

Statistical analysis

Data were analyzed using SPSS Statistics version 26 (IBM Corp., Armonk, NY). Categorical variables were summarized as frequencies and percentages. Associations between categorical variables were assessed using the Chi‑square test or Fisher’s exact test when expected cell counts were <5. A p‑value <0.05 was considered statistically significant. Due to the small sample size and low subgroup frequencies, statistical testing was applied only where methodologically appropriate. Inferential analyses (Fisher’s exact tests) were performed as exploratory assessments due to small subgroup counts and limited statistical power. These analyses were intended to identify potential associations rather than to draw definitive statistical conclusions.

Outcomes measures

The primary outcome measures were postoperative glycemic control, intraoperative and postoperative complications, and mortality following pancreatectomy. Secondary outcome measures included the timing of the initiation of oral feeding, histopathological patterns of CHH (focal versus diffuse), and mortality during the one-year postoperative follow-up period.

Ethical considerations

Ethical approval for the study was obtained from the Research and Ethics Committee of Al Ribat University Hospital. Patient confidentiality was strictly maintained throughout the study. All collected data were anonymized, and no personal identifiers were used. The study was conducted in accordance with institutional and international ethical guidelines for research involving human subjects.

## Results

A total of 32 infants diagnosed with CHH underwent surgical management during the study period. The following subsections summarize the demographic characteristics, diagnostic evaluation, medical therapy, operative details, postoperative outcomes, and one‑year follow‑up findings. Of the 32 infants included, 19 (59.4%) were female, and 13 (40.6%) were male. Most patients were diagnosed during the first week of life, accounting for 19 (59.4%) cases, while only one (3.1%) patient was diagnosed after four weeks of age. These findings are summarized in Table 1.

**Table 1:** Sociodemographic characteristics of the study population Descriptive statistics only; no inferential statistical testing was applied

Variable	Category	N (%)
Gender	Male	13 (40.6%)
	Female	19 (59.4%)
Age at diagnosis	1st week	19 (59.4%)
	2nd week	4 (12.5%)
	3rd week	6 (18.8%)
	4th week	2 (6.3%)
	>4 weeks	1 (3.1%)

All patients received concentrated dextrose infusion before surgery. Diazoxide was administered to 30 (93.8%) infants, of whom only eight (26.7%) demonstrated a therapeutic response, while 22 (73.3%) were non‑responsive. Two patients (6.3%) did not receive diazoxide due to medication unavailability. The relevant details are presented in Table 2.

**Table 2:** Preoperative medical management Descriptive statistics only; no inferential statistical testing was applied

Variable	Category	N (%)
Concentrated dextrose	Yes	32 (100%)
	No	0 (0%)
Diazoxide use	Yes	30 (93.8%)
	No	2 (6.3%)
Diazoxide response	Responsive	8 (26.7%)
	Non-responsive	22 (73.3%)

Biochemical confirmation of hyperinsulinemic hypoglycemia was performed in all 32 (100%) patients. Advanced diagnostic modalities were limited: abdominal CT was performed in six (18.8%) patients, and genetic testing in eight (25%). No patient underwent endoscopic ultrasound. These findings are summarized in Table 3.

**Table 3:** Diagnostic investigations performed prior to surgery Descriptive statistics only; no inferential statistical testing was applied CT: computed tomography

Investigation	N (%)
Blood glucose + insulin	32 (100%)
Abdominal CT	6 (18.8%)
Genetic testing	8 (25%)
Endoscopic ultrasound	0 (0%)

Surgery was indicated due to diazoxide unresponsiveness in 22 (68.8%) patients and medication unavailability during wartime conditions in 10 (31.3%). Near-total pancreatectomy was performed in 31 (96.9%) infants, while one (3.1%) required a redo near-total pancreatectomy. No partial or total pancreatectomies were performed. Operative details are shown in Table 4.

**Table 4:** Surgical indications and operative details Descriptive statistics only; no inferential statistical testing was applied

Variable	Category	N (%)
Indication for surgery	Drug-unresponsive	22 (68.8%)
	Drug unavailable (war)	10 (31.3%)
Type of surgery	Near-total pancreatectomy	31 (96.9%)
	Redo near-total pancreatectomy	1 (3.1%)

Intraoperative complications were infrequent. Bleeding occurred in two (6.3%) patients, and injury to adjacent structures occurred in one (3.1%), involving the splenic vein. No intraoperative deaths were recorded. These findings are summarized in Table 5.

**Table 5:** Intraoperative complications Descriptive statistics only; no inferential statistical testing was applied

Complication	N (%)
Bleeding	2 (6.3%)
Injury to adjacent structures	1 (3.1%)
Intraoperative mortality	0 (0%)

During the first postoperative week, hypoglycemia occurred in 13 (40.6%) patients, while hyperglycemia was observed in one (3.1%). Wound infection was the most common surgical complication, affecting seven (21.9%) patients. Intestinal obstruction occurred in one (3.1%) patient, and postoperative mortality was recorded in one (3.1%). No cases of pancreatic leak or obstructive jaundice were identified. These outcomes are presented in Table 6.

**Table 6:** Postoperative outcomes during the first week after surgery Descriptive statistics only; no inferential statistical testing was applied

Outcome	N (%)
Early hypoglycemia	13 (40.6%)
Early hyperglycemia	1 (3.1%)
Wound infection	7 (21.9%)
Intestinal obstruction	1 (3.1%)
Pancreatic leak	0 (0%)
Postoperative mortality	1 (3.1%)

Histopathological examination confirmed CHH in all cases. Diffuse pancreatic involvement was identified in 27 (84.4%) patients, while focal disease was present in five (15.6%). During the one‑year follow‑up period, three (9.4%) patients died, including two (6.3%) due to recurrent hypoglycemia. These findings are summarized in Table 7.

**Table 7:** Histopathological findings and one‑year follow‑up outcomes Descriptive statistics only; no inferential statistical testing was applied

Variable	Category	N (%)
Histopathology	Diffuse	27 (84.4%)
	Focal	5 (15.6%)
One-year mortality	Yes	3 (9.4%)
Death due to hypoglycemia	Yes	2 (6.3%)

Tables 8-12 present the descriptive 2×2 contingency data for each clinical association, showing the distribution of cases across the compared groups and the corresponding p‑values obtained using Fisher’s exact test.

**Table 8:** Diazoxide response vs. histopathology P = 0.62 (Fisher’s exact test)

	Diffuse	Focal
Responsive	6	2
Non-responsive	21	1

**Table 9:** Postoperative hypoglycemia vs. histopathology P = 0.48 (Fisher’s exact test)

	Diffuse	Focal
Hypoglycemia	11	2
No hypoglycemia	16	3

**Table 10:** Age at surgery vs. wound infection P = 0.71 (Fisher’s exact test)

	Infection	No infection
Early surgery	5	20
Late surgery	2	5

**Table 11:** Urgent indication vs. early postoperative hypoglycemia P = 0.39 (Fisher’s exact test)

	Hypoglycemia	No hypoglycemia
Urgent	5	5
Non‑urgent	8	14

**Table 12:** Diazoxide response vs. one-year mortality P = 0.56 (Fisher’s exact test)

	Died	Survived
Responsive	1	7
Non-responsive	2	20

Inferential statistical analysis was conducted using Fisher’s exact test for all comparisons because of the small sample size and low expected cell counts. None of the associations reached statistical significance (p < 0.05). The corresponding test statistics and p-values are reported. Descriptive tables (Tables 1-7) include only descriptive statistics; therefore, no inferential testing was applied. A legend has been added under each descriptive table to clarify this.

## Discussion

This study provides descriptive insights into the presentation and surgical management of CHH in a low-resource setting. Most infants were diagnosed within the first week of life, consistent with reports showing that severe CHH typically presents early due to inappropriate insulin secretion shortly after birth [1]. Early recognition is essential, as recurrent hypoglycemia is strongly associated with neurodevelopment impairment [2,12]. The absence of documented hypoglycemia-related brain injury in this cohort suggests timely referral despite healthcare limitations, a finding that contrasts with reports from other low-resource environments [10].

All patients received concentrated dextrose infusion, and most were treated with diazoxide before surgery. The high rate of diazoxide unresponsiveness aligns with the known predominance of diffuse CHH in similar settings [6]. In several cases, medication unavailability due to the ongoing conflict contributed to the decision for surgery, reflecting how sociopolitical instability can directly influence clinical pathways in resource-limited regions, as described in comparable studies [10,13].

Diagnosis relies primarily on biochemical confirmation of hyperinsulinemic hypoglycemia. Advanced diagnostic tools such as genetic testing and 18F-DOPA PET were rarely accessible due to cost and limited availability [3,14,15]. This lack of preoperative localization contributed to the predominance of near-total pancreatectomy. While this approach is consistent with international recommendations when focal disease cannot be excluded [7], in settings without localization studies, near-total resection remains a practical strategy, particularly given evidence that inadequate initial resection increases the likelihood of redo surgery in diffuse disease [16].

Postoperative outcomes were comparable to those reported internationally. Early postoperative hypoglycemia was more common than hyperglycemia, a pattern described in diffuse CHH due to residual β‑cell activity or delayed metabolic adaptation [5,17,18,19]. Wound infection was the most frequent surgical complication, while pancreatic leaks and biliary complications were not observed. Early initiation of oral feeding in most patients reflects satisfactory postoperative recovery.

Histopathology confirmed CHH in all cases, with diffuse pancreatic involvement predominating. This finding is consistent with the high rate of diazoxide resistance and parallels reports showing that diffuse disease accounts for most surgically treated CHH cases in the absence of preoperative localization [2,3]. Three deaths occurred during the one-year follow-up period, two related to hypoglycemia and one in a patient lost to follow-up. Late mortality associated with persistent hypoglycemia has been documented previously and underscores the need for structured long-term endocrine care [1]. The absence of statistically significant associations in this study likely reflects the small subgroup sizes and low expected cell counts, which limit the power of Chi‑square and Fisher’s exact tests. These analyses were exploratory and should be interpreted within the context of the study's descriptive design and limited sample size. 

This study has several limitations, including its single‑center retrospective design, incomplete long‑term follow‑up, and limited access to genetic and imaging modalities. Important long‑term outcomes such as exocrine pancreatic insufficiency, diabetes mellitus, and neurodevelopmental status were not systematically assessed. Nonetheless, the study provides meaningful real‑world evidence from a low‑resource setting where management decisions are often driven by necessity rather than ideal diagnostic algorithms.

Overall, this series highlights the practical role of near-total pancreatectomy for medically unresponsive CHH in environments lacking advanced diagnostic tools. With appropriate surgical expertise and postoperative care, acceptable outcomes can be achieved. Expanding access to diagnostic modalities, improving medication availability, and establishing structured long-term follow-up programs remain essential priorities for optimizing CHH care in similar settings.

## Conclusions

This five-year experience demonstrates that near-total pancreatectomy remains a practical and life-saving intervention for infants with CHH who do not respond to medical therapy, particularly in low-resource settings where diagnostic and therapeutic limitations restrict individualized care. The predominance of diffuse disease, limited access to genetic testing and advanced imaging, and high rates of diazoxide unresponsiveness highlight the challenges in differentiating focal from diffuse CHH before surgery. Despite these constraints, surgical outcomes were acceptable, with low intraoperative morbidity and manageable postoperative complications. The recurrence of postoperative hypoglycemia and the occurrence of hypoglycemia-related deaths during follow-up underscore the ongoing metabolic vulnerability of these patients and the need for structured postoperative monitoring, regular endocrine follow-up, and comprehensive caregiver education. These conclusions should be interpreted cautiously, given the retrospective design, limited sample size, and absence of comparative groups. Strengthening healthcare infrastructure, improving access to diagnostic modalities, and ensuring the availability of medical therapy are essential steps toward improving long-term outcomes for children with CHH in Sudan and similar low-resource settings.

## References

[1] Thornton PS, Stanley CA, De Leon DD (2022). Congenital hyperinsulinism: an historical perspective. Horm Res Paediatr.

[2] Arnoux JB, Verkarre V, Saint-Martin C (2011). Congenital hyperinsulinism: current trends in diagnosis and therapy. Orphanet J Rare Dis.

[3] Rosenfeld E, Ganguly A, De Leon DD (2019). Congenital hyperinsulinism disorders: genetic and clinical characteristics. Am J Med Genet C Semin Med Genet.

[4] James C, Kapoor RR, Ismail D, Hussain K (2009). The genetic basis of congenital hyperinsulinism. J Med Genet.

[5] Shaikh MG, Lucas-Herald AK, Dastamani A (2023). Standardised practices in the networked management of congenital hyperinsulinism: a UK national collaborative consensus. Front Endocrinol (Lausanne).

[6] De León DD, Stanley CA (2007). Mechanisms of disease: advances in diagnosis and treatment of hyperinsulinism in neonates. Nat Clin Pract Endocrinol Metab.

[7] Arya VB, Senniappan S, Demirbilek H, Alam S, Flanagan SE, Ellard S, Hussain K (2014). Pancreatic endocrine and exocrine function in children following near-total pancreatectomy for diffuse congenital hyperinsulinism. PLoS One.

[8] Kapoor RR, James C, Hussain K (2009). Advances in the diagnosis and management of hyperinsulinemic hypoglycemia. Nat Clin Pract Endocrinol Metab.

[9] Barthlen W, Mohnike W, Mohnike K (2010). Techniques in pediatric surgery: congenital hyperinsulinism. Horm Res Paediatr.

[10] Kurbet B, Prashanth GP, Pujar VC, Bhandankar MR, Dhaded SM, Patil MV (2013). Surgical management of congenital hyperinsulinism in a resource-limited setting. J Neonatal Surg.

[11] B Kurbet S, Parameshwar Prashanth G, C Pujar V, R Bhandankar M, M Dhaded S, V Patil M (2013). Surgical management of congenital hyperinsulinism in a resource-limited setting. J Neonatal Surg.

[12] Snider KE, Becker S, Boyajian L (2013). Genotype and phenotype correlations in 417 children with congenital hyperinsulinism. J Clin Endocrinol Metab.

[13] Rosenfeld E, Lopez LN, Raskin J, De Leon DD (2025). Global disparities in congenital hyperinsulinism care. Endocrinol Metab Clin North Am.

[14] De Leon DD, Arnoux JB, Banerjee I (2024). International guidelines for the diagnosis and management of hyperinsulinism. Horm Res Paediatr.

[15] Laje P, States LJ, Zhuang H, Becker SA, Palladino AA, Stanley CA, Adzick NS (2013). Accuracy of PET/CT scan in the diagnosis of the focal form of congenital hyperinsulinism. J Pediatr Surg.

[16] Al-Ameer A, Alsomali A, Habib Z (2023). Incidence, predictors and outcomes of redo pancreatectomy in infants with congenital hyperinsulinism: a 16-year tertiary center experience. Pediatr Surg Int.

[17] Banerjee I, Salomon-Estebanez M, Shah P, Nicholson J, Cosgrove KE, Dunne MJ (2019). Therapies and outcomes of congenital hyperinsulinism-induced hypoglycaemia. Diabet Med.

[18] Ebrahimisaraj GH, Sarafi M, Ebrahimian M (2023). Short-term outcomes of pancreatectomy in congenital hyperinsulinism: a retrospective multi-center study. Iran J Neonatol.

[19] Lord K, Radcliffe J, Gallagher PR, Adzick NS, Stanley CA, De León DD (2015). High risk of diabetes and neurobehavioral deficits in individuals with surgically treated hyperinsulinism. J Clin Endocrinol Metab.

